# A single-blind, randomized, crossover study on the efficacy of icatibant for sweating-induced dermal pain (icatibant for sweating-induced dermal pain)

**DOI:** 10.1097/MD.0000000000033971

**Published:** 2023-06-09

**Authors:** Shunsuke Takahagi, Michihiro Hide, Yumi Aoyama, Atsushi Fukunaga, Hiroyuki Murota

**Affiliations:** a Department of Dermatology, Graduate School of Biomedical & Health Sciences, Hiroshima University, Minami-ku, Hiroshima, Japan; b Department of Dermatology, Hiroshima City Hiroshima Citizens Hospital, Naka-ku, Hiroshima, Japan; c Department of Dermatology, Kawasaki Medical School Hospital, Kurashiki, Japan; d Division of Dermatology, Department of Internal Related, Kobe University Graduate School of Medicine, Chuo-ku, Kobe, Japan; e Department of Dermatology, Division of Medicine for Function and Morphology of Sensory Organs, Faculty of Medicine, Osaka Medical and Pharmaceutical University, Takatsuki, Osaka, Japan; f Department of Dermatology and Allergy, Nagasaki University Hospital, Nagasaki, Japan.

**Keywords:** bradykinin, bradykinin B2 receptor, dermal pain, icatibant, sweating

## Abstract

Severe dermal pain triggered by sweating stimuli, such as bathing, exercise, and mental stress, significantly affects patients’ daily lives. The pathomechanism underlying the sweating-induced dermal pain remains poorly understood and there exists no standard treatment for such pain. This study aims to evaluate the effectiveness of icatibant as an analgesic, a bradykinin B2 receptor antagonist, in treating sweating-induced dermal pain, and to establish the role of bradykinin in pain induction.

**Methods/design:**

A multicenter, exploratory, crossover, single-blinded, placebo-controlled randomized, comparative study will be conducted to evaluate the efficacy of subcutaneous icatibant injection (30 mg) in treating sweating-induced dermal pain. Ten patients will be enrolled and assigned randomly in a 1:1 ratio to either the icatibant-placebo or placebo-icatibant groups. The primary endpoint is the change in the visual analog scale scores for dermal pain induced by thermal load before and after treatment with icatibant or placebo. Secondary endpoints include changes in the duration of dermal pain, blood and plasma histamine levels, serum angiotensin-converting enzyme levels, and histological evaluation of skin tissue samples at the site of dermal pain.

**Discussion:**

The effectiveness of icatibant against sweating-induced dermal pain would provide clear evidence for the involvement of the bradykinin-bradykinin B2 receptor pathway in the pathogenesis of this condition. This finding may contribute to a better understanding of the underlying mechanisms of dermal pain associated with sweating stimuli and has the potential to improve patients’ quality of life by suggesting potential treatment options, specifically, using drugs that inhibit bradykinin or suppress its production.

## 1. Introduction

In a certain population of individuals, the exposure to sweating stimuli such as bathing, exercise, or mental stress, leads to severe dermal pain.^[1–3]^ The sweating-induced dermal pain may occur alone or more frequently be accompanied by transient eruptions and/or generalized hypohidrosis. When accompanied by such symptoms, the condition can also be referred to as cholinergic urticaria^[2]^ or acquired idiopathic generalized anhidrosis.^[3,4]^ Such dermal pain predominantly affects young males in their teens and twenties^[1]^ and manifests immediately upon exposure to sweating stimuli, lasting typically for 5 to 10 minutes at a maximum visual analog scale (VAS) level of 100 mm.^[1]^ Although short lived, the pain is severe and significantly disrupts patients daily lives, resulting in absenteeism or withdrawal from school or work.^[1]^ Consequently, there is a substantial unmet need to understand the underlying mechanisms and develop effective treatments.

Despite numerous attempts to treat sweating-induced dermal pain, no standard treatment has been established. Common nonsteroidal anti-inflammatory and analgesic drugs demonstrate little effect. As the condition frequently presents with transient eruptions and/or generalized hypohidrosis, antihistamines and systemic corticosteroids have been tested based on their effectiveness against cholinergic urticaria and acquired idiopathic generalized anhidrosis.^[1,3]^ However, these treatments fail to fully alleviate pain triggered by such stimuli.^[1,5,6]^ The use of psychotropic drugs and neuropathic pain medications has also been explored, but their efficacy varies among patients.^[1,5,6]^ The absence of a suitable animal model for sweating-induced dermal pain complicates the evaluation of therapeutic agents.

The precise pathomechanisms underlying sweating-induced dermal pain remain elusive. Short duration of the pain following sweating stimuli suggests the involvement of endogenous pain-producing substances with short lifetimes, such as histamine and bradykinin in the pathogenesis of sweating-induced dermal pain. High levels of histamine in sweat and elevated plasma histamine levels after thermal sweating in patients with dermal pain compared to healthy subjects^[1]^ support the potential involvement of histamine. However, antihistamines alone do not provide sufficient analgesia. Alternatively, bradykinin may serve as a mediator, inducing dermal pain triggered by sweating stimuli. Kininogen and kallikrein, which are involved in bradykinin production, are reportedly present in sweat glands.^[7]^ Additional study revealed that eccrine sweat gland stimulation by thermal load activated the bradykinin-forming enzyme in the sweat and the formation of bradykinin in the skin.^[8]^ The bradykinin-forming enzyme is shown to be tissue kallikrein, and contribute to generate Lys-bradykinin which acts on bradykinin B2 receptor.^[9,10]^ Localization of kallikrein is detected in luminal ductal cells and in the peripheral rim of secretory coil segments.^[9]^ The stimulation of bradykinin B2 receptor is reported not to be associated with thermally- and pilocarpine-induced sweating itself^[11]^; however, human sweat glands respond to bradykinin stimulation to increase cyclic AMP for flexible control of sweat gland function.^[12]^ Although the role of bradykinin-forming activity detected in sweat glands remains unclear, these suggest that increased bradykinin production in the skin during sweating stimulation could contribute to dermal pain.

This study aims to evaluate the analgesic effect of icatibant, a bradykinin B2 receptor antagonist, on patients experiencing sweating-induced dermal pain and to demonstrate a role of bradykinin in the pathomechanism of dermal pain. Its results are expected to contribute to the development of novel treatments for sweating-induced dermal pain targeting bradykinin and the kallikrein-kinin pathway.

## 2. Methods/design

### 2.1. Study design

This is a multicenter, exploratory, crossover, single-blind, placebo-controlled, and randomized comparative study.

### 2.2. Study setting

Study participants include patients with sweating-induced dermal pain who visit Hiroshima University Hospital directly or are referred to Hiroshima University Hospital by collaborating institutions (Kawasaki Medical School Hospital, Kobe University Hospital, and Nagasaki University Hospital) to seek new treatment options. After obtaining consent from study participants at Hiroshima University Hospital, eligibility will be confirmed based on the inclusion and exclusion criteria. Upon enrollment, participants will be assigned to 1 of 2 groups (Icatibant-placebo group [Group A] or Placebo-icatibant group [Group B]). Treatment drugs will be administered in a single-blinded crossover manner, and dermal pain induced by thermal load will be evaluated before and after administration. To explore related mediators, blood and plasma histamine levels and plasma angiotensin-converting enzyme (ACE) levels will also be measured before and after thermal load.

### 2.3. Inclusion criteria

1. Male and female subjects aged ≥ 18 years and ≤ 50 years at the time of obtaining consent.
2. Patients with severe dermal pain induced by thermal sweating stimuli (e.g., bathing), regardless of the presence or absence of cholinergic urticaria and hypohidrosis.
3. Patients who have had symptoms of dermal pain for more than 3 months.
4. Patients with a dermal pain degree ≥ VAS 60 mm in their daily life during the 2 weeks before obtaining consent.
5. Patients who have not changed drugs* that may alleviate symptoms during the 2 weeks before obtaining consent and have no plans to change drugs until the end of this study (including injections, oral medications, and topical medications). The asterisk refers to antihistamines, H2 receptor antagonists, leukotriene receptor antagonists, antipyretic analgesics, narcotics, antipsychotics, and drugs acting on the central and peripheral nervous systems.
6. Patients whose consent for participation in this study can be obtained in writing.

### 2.4. Exclusion criteria

1. Patients with other causes for dermal pain.
2. Patients with atopic dermatitis, Sjögren syndrome, hypothyroidism, Fabry disease, autonomic nervous system disorders, or drug-induced anhidrosis. To exclude these diseases, SS-A antibodies, SS-B antibodies, FT3, FT4, and TSH will be measured, and subjects with abnormal values will be excluded. In addition, α-galactosidase activity will be measured in patients who experience pain in their hands and feet, not related to temperature elevation, and hypohidrosis that appeared before school age. Genetic testing of α-galactosidase may need to be performed in women. Patients with these abnormalities would be excluded from the study.
3. Patients with a history of allergy to icatibant.
4. Patients who are pregnant or breastfeeding.

### 2.5. Randomization and allocation concealment

**Figure 1.**
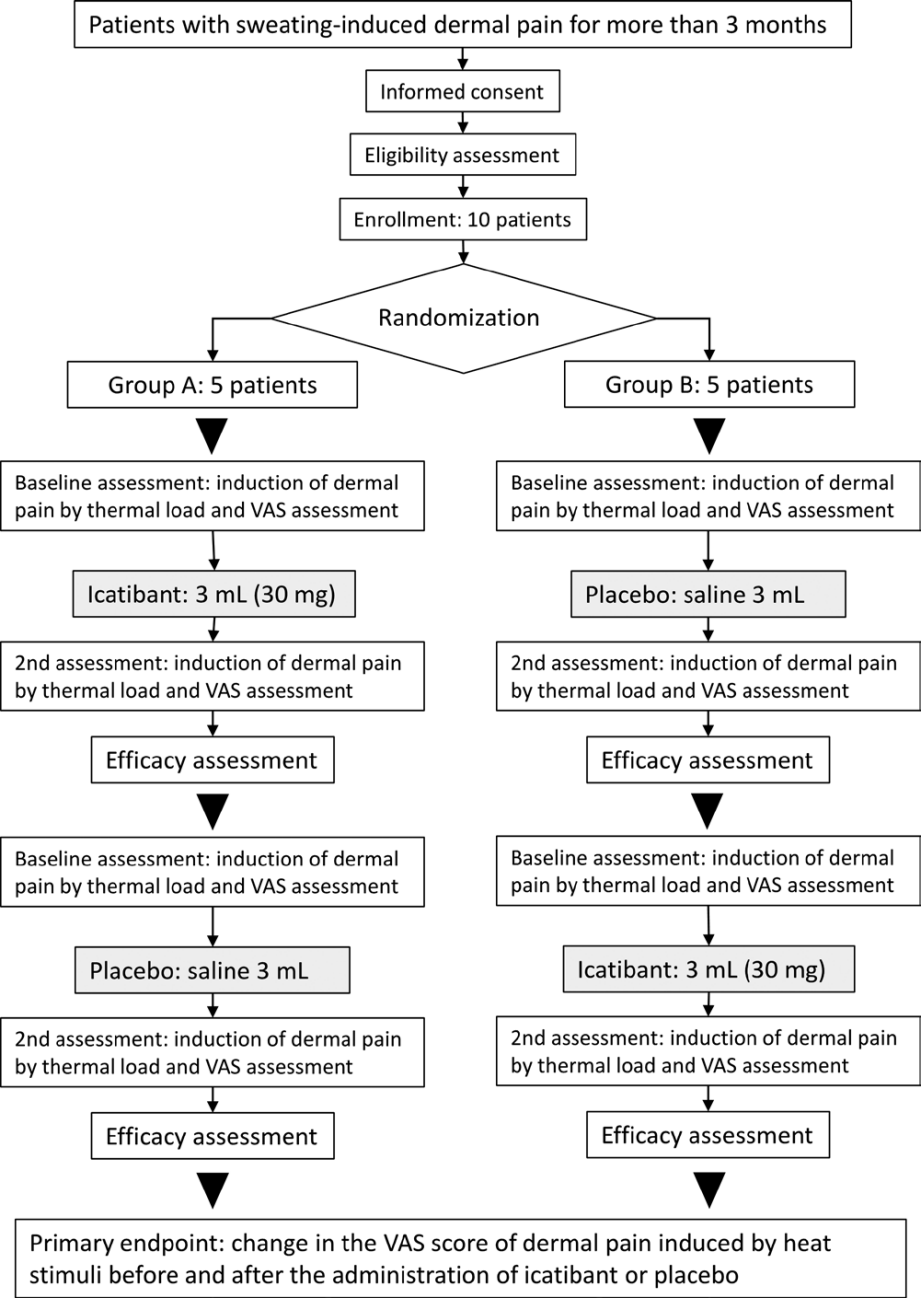
Flowchart of the study design.

The study procedures and schedule are shown in Figure 1 and Table 1, respectively. Allocation of study participants to each treatment group will be performed through a central registry. According to a predesigned computer-generated random assignment table, participants will be assigned in a 1:1 ratio to either Icatibant-placebo group (Group A) or Placebo-icatibant group (Group B).

**Table 1:**
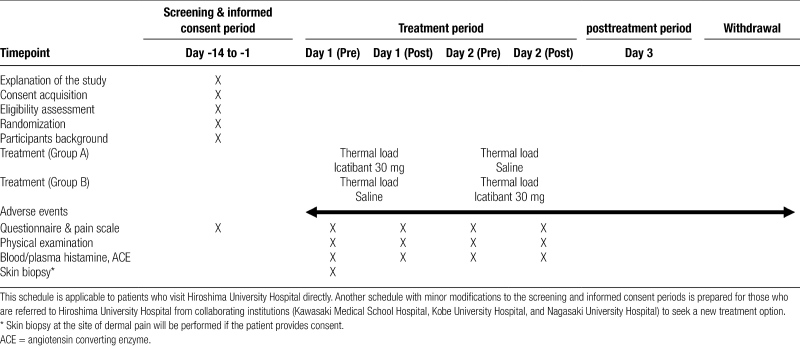
Intervention and assessment schedule.

### 2.6. Interventions

Icatibant (30 mg) and placebo (saline, 3 mL) will be subcutaneously injected in a defined order in a crossover manner. Dermal pain induced by thermal load will be assessed before and after administration of both drugs. For blinding, study participants wear an eye mask when receiving the drugs. The investigator will ensure that the study participant has properly donned the eye mask before preparing the drugs and giving them to the participants. After the used syringes are discarded, the participant will be allowed to remove the eye mask. The study will be conducted over 3 consecutive days under hospitalization.

#### 2.6.1. [Day 1].

After collecting patients’ background data, both lower legs will be subjected to thermal load by placing them in warm water (~43°C) for 30 minutes to induce dermal pain.^[13]^ Scheduled examinations and blood sampling will be performed before and after thermal loading. The examinations include VAS for pain, distribution and duration of dermal pain, VAS for itching, and presence or absence of eruption. If unbearable dermal pain occurs during heating, the thermal load will be discontinued, and examinations and blood sampling will be performed. Icatibant (30 mg) or placebo (saline, 3mL) will be subcutaneously injected at least 90 minutes after the first thermal load or 30 minutes after the skin symptoms resolved, whichever occurs later. The second thermal load starts 45 ± 15 min after the drug administration, and the second scheduled examinations and blood sampling will be performed before and after the second thermal load.

#### 2.6.2. [Day 2].

The same procedure as on Day 1 will be followed to administer the drug and conduct the (3rd and 4th) thermal load before and after drug administration. The placebo (saline) will be subcutaneously injected into patients who receive icatibant on Day 1, while icatibant to those who receive placebo on Day 1. A washout period of ≥ 9 h will be allowed between drug administration on Day 1 and 2. The period was established as 5 times the half-life (T1/2 1.7 hours) of icatibant^[14]^ or longer.

#### 2.6.3. [Day 3].

Subjects will be checked for adverse events.

### 2.7. Outcomes

#### 2.7.1. Primary endpoint.

The primary endpoint is the change in the VAS score for thermally-induced dermal pain before and after treatment with icatibant or placebo.

#### 2.7.2. Secondary endpoints.

1. Changes in the duration of dermal pain induced by thermal load before and after administration of icatibant or placebo.
2. Blood samples will be collected before and after treatment with icatibant or placebo to measure blood and plasma histamine, and serum ACE levels.
3. In patients who consent to a skin biopsy, a skin tissue sample will be obtained to histologically evaluate the pathomechanism of dermal pain. In some patients, a prior skin biopsy may have been performed at the site of dermal pain beforehand in the past clinical practice. If the conditions for obtaining skin samples are consistent with those in this study, the previous specimens would be evaluated to avoid performing another biopsy with patients physical burden.

#### 2.7.3. Adverse events.

Safety concerns in this study are assumed to be associated with the administration of icatibant and the induction of dermal pain by thermal stimuli. Safety will be assessed by monitoring adverse events, physical findings, and changes in vital signs. If an adverse event is clinically suspected, physicians will conduct a thorough examination and safety evaluation.

### 2.8. Statistical methods

#### 2.8.1. Sample size.

Target number of patients is 5 patients in the icatibant-placebo group (Group A), and 5 patients in the placebo-icatibant group (Group B). Considering the minimum number of patients to achieve the study objectives and the rarity of sweating-induced dermal pain, total 10 eligible patients will be enrolled and evaluated in this proof-of-concept study.

#### 2.8.2. Target population for analysis.

The population to be analyzed is defined as follows.

1. Full analysis set (FAS): The FAS will comprise all study subjects who underwent randomization, excluding the following study subjects: cases that do not meet the eligibility criteria (e.g., diagnosed with another disease, violating objectively determinable inclusion or exclusion criteria), cases that have never received any study treatment after randomization, and cases with no measurements made after randomization.
2. Per protocol set (PPS): The PPS consisted of the FAS population, excluding the following study subjects: cases with unavailable measurements of the primary endpoint and cases with serious protocol violations (e.g., misassignment, failure to meet eligibility, use of prohibited medications, and noncompliance with medications).

#### 2.8.3. Analysis of the primary endpoint.

The primary endpoint of the study is the change in the VAS scores for dermal pain induced by thermal load before and after treatment with icatibant or placebo. The FAS will be used as the analysis population to compare the changes in VAS scores between the 2 treatments at a significance of *P* < .05 (2-tailed). Paired t-tests will be performed to determine the significance of differences.

#### 2.8.4. Analysis of the secondary endpoints.

The FAS will be the population to be analyzed. A comparison of the change in duration of dermal pain induced by thermal load will be examined at a significance of *P* < .05 between the icatibant and placebo treatment phases. Additionally, changes of blood and plasma histamine levels and serum ACE levels before and after treatment will be analyzed at a significance of *P* < .05 between both treatment phases. A secondary analysis will be performed using the PPS as the analysis population by the same statistical methods. Skin samples will be collected for the histological evaluation of dermal pain. In some patients, a prior skin biopsy may have been performed at the site of dermal pain in conditions consistent with those in this study. The previous specimens will be evaluated in those cases.

#### 2.8.5. Safety assessments.

In the population of study subjects who receive at least 1 study treatment, the number and incidence of adverse events and the number of subjects suffering from adverse events will be investigated in each phase of the icatibant and placebo treatment.

### 2.9. Monitoring and auditing

The principal investigator will designate monitoring personnel to monitor the study to ensure that the study is being conducted safely and in accordance with both the protocol and the Clinical Research Act, and that data are being collected accurately. The person in charge of monitoring will report the monitoring results to the principal investigator. No audit is applicable to this study.

### 2.10. Harms

Adverse event is defined as illness, disability, death, or infection suspected to be attributed to conducting the study, including unintended physical signs, clinically significant changes in laboratory data, and worsening of symptoms or complications. In the event of harms, the principal investigator or sub-investigator will take appropriate actions to ensure the safety of study participants, including giving treatments and discontinuing administration of the research drugs to study participants.

## 3. Discussion

Sweating-induced dermal pain is triggered by daily events, such as bathing, exercise, and mental stress.^[1–3]^ It is transient but brings severe and heavy impacts on patients lives. The pathomechanism of sweating-induced dermal pain remains unclear, and no standard treatment has been established. Treatments may include antihistamines, steroid pulse therapy, psychotropic drugs, and the Ca^2+^ channel α2δ ligand, but their effectiveness is limited and varies among patients.^[1]^

Bradykinin is an endogenous substance that causes pain.^[15]^ Since predominant kinin-producing activity is detected in the skin during thermal sweating, bradykinin may play a role in pain during sweating stimulation.^[7–10]^ Icatibant, a bradykinin B2 receptor antagonist, has been approved for the treatment of acute attacks of hereditary angioedema,^[14,16]^ but been off-label for dermal pain associated with sweating stimuli. Subcutaneous administration of 30 mg icatibant results in a rapid increase in blood icatibant levels (Tmax 0.63 hour) and effectively inhibits bradykinin activity.^[14]^ If a subcutaneous injection of icatibant effectively reduces sweating-induced dermal pain, it would provide clear evidence for the involvement of the bradykinin-bradykinin B2 receptor pathway in this condition.

This study is a crossover, single-blind, placebo-controlled, randomized comparative study. Due to the rarity of patients with sweating-induced dermal pain, the number of patients capable to be enrolled in the study is limited. Moreover, the subjective nature of pain perception makes it difficult to compare pain levels across patients. To minimize these limitations, a crossover design is employed. Furthermore, to account for the potential placebo effect on dermal pain due to expectations of drug efficacy, the present study is designed as a single-blinded, placebo-controlled, randomized study.

The icatibant dose setting is based on its efficacy and safety. A pharmacodynamic analysis estimated the tolerated dosage needed to reduce bradykinin-induced reactions to be 0.4 mg/kg,^[14]^ that is, 30 mg for an adult. Moreover, the approved dose of icatibant for hereditary angioedema is 30 mg/dose and has no frequent harmful events, except for transient local injection site reactions.^[14,16]^ In this study, the same dosage of 30 mg used for hereditary angioedema is set for dermal pain. Since icatibant is typically administered as a single dose for hereditary angioedema attacks,^[14]^ the treatment in this study also includes a single administration of icatibant or placebo.

The primary endpoint is to assess the effectiveness of icatibant in treating sweating-induced dermal pain. Pain will be evaluated using a VAS score by inducing dermal pain with a thermal load before and after icatibant administration. This approach has been chosen due to the importance of evaluating pain in patients with the dermal pain and the subjective nature of pain perception. To induce dermal pain, both lower legs will be placed in warm water (~43°C) for 30 minutes.^[13]^ This condition of loading is enough to induce sweating in healthy subjects, commonly experienced in daily life, and thus, non-excessive in terms of intensity and duration. The VAS score, a widely used pain rating scale, will be employed to measure the intensity of the pain.

Blood histamine and ACE levels will be measured as biomarkers. Similar to bradykinin, histamine causes skin pain and may be associated with dermal pain.^[1]^ ACE is an enzyme involved in the kallikrein/kinin system that degrades and inactivates bradykinin.^[15]^ By analyzing the dynamics of these molecules before and after thermal sweating, we will evaluate the relationship between dermal pain occurrence, histamine in circulation, and the kallikrein-kinin system. Additionally, to explore the pathomechanism of dermal pain, we will examine histological changes of the eccrine sweat glands at the site of dermal pain in patients who provide consent for a skin biopsy. Histological analyses focus on pore obstruction, reduction in the number and atrophy of sweat glands, and surrounding inflammatory infiltration.^[1,17–19]^

A limitation of this study is that a small number of patients are planned to be enrolled. Since this preliminary study is conducted for proof of concept, only ten patients will participate to explore the effect of blocking kinin-bradykinin pathway to sweating-induced dermal pain. Moreover, the efficacy of icatibant in this study does not necessarily imply that the use of icatibant is a practical treatment for the pain due to its short effective duration of action. According to pharmacodynamic analysis, icatibant exerts its effectiveness for only 5.5 to 6 hours.^[14]^ This indicates that multiple administrations in a day are necessary for its clinical application. However, this is not realistic because icatibant is administered subcutaneously rather than orally and is expensive, thereby posing a high-cost burden for patients. Alternatively, this study may pave the way for novel treatments of dermal pain using kallikrein inhibitors. Kallikrein produces bradykinin by acting on high-molecular-weight kininogens; therefore, kallikrein inhibitors suppress the subsequent bradykinin-mediated responses.^[15]^ Kallikrein inhibitors berotralstat^[20]^ and lanadelumab^[21]^ are an oral formulation and a subcutaneous formulation with a considerably longer duration of effect, respectively.

Overall, this study contributes to a better understanding of the underlying mechanisms of sweating-induced dermal pain and has the potential to improve the quality of life for patients experiencing this debilitating condition by suggesting potential treatment options.

## 4. Trial status

The study was approved on December 22, 2020. Participant recruitment will begin on July 8, 2021. The expected date of completion is the end of December 2024. Central ethical approval was obtained from the Hiroshima University Certified Review Board (reference approval number: CRB6180006). The protocol and progress have been registered in Japan Registry of Clinical Trials (jRCT), jRCTs061210021 since July 8, 2022. Protocol version is v1.3 as on February, 2022.

## Acknowledgments

This study is conducted with the free supply of the research drug (Firazyl® subcutaneous injection 30 mg syringe) from Takeda Pharmaceuticals International AG, a subsidiary of Takeda Pharmaceutical Company Limited. It is supported by Clinical Research Center in Hiroshima. We offer a special thank you also to all the staff involved in this study.

## Author contributions

**Conceptualization:** Shunsuke Takahagi, Michihiro Hide.

**Funding acquisition:** Shunsuke Takahagi, Michihiro Hide.

**Investigation:** Shunsuke Takahagi, Yumi Aoyama, Atsushi Fukunaga, Hiroyuki Murota.

**Methodology:** Shunsuke Takahagi, Michihiro Hide, Yumi Aoyama, Atsushi Fukunaga, Hiroyuki Murota.

**Resources:** Shunsuke Takahagi, Michihiro Hide.

**Supervision:** Michihiro Hide.

**Visualization:** Shunsuke Takahagi.

**Writing – original draft:** Shunsuke Takahagi.

**Writing – review & editing:** Michihiro Hide, Yumi Aoyama, Atsushi Fukunaga, Hiroyuki Murota.
